# Metagenomic Analysis Identifying a Rare *Leishmania* Infection in an Adult With AIDS

**DOI:** 10.3389/fcimb.2021.764142

**Published:** 2021-12-15

**Authors:** Pingping Song, Shuai Chen, Xiaoyu Tan, Yanjun Gao, Juanjuan Fu, Zhiqing You, Chengtan Wang, Qigang Zhao, Feng Pang

**Affiliations:** ^1^ Department of Clinical Laboratory, Liaocheng People’s Hospital, Liaocheng, China; ^2^ Department of Clinical Laboratory, Liaocheng Third People’s Hospital, Liaocheng, China

**Keywords:** leishmaniasis, *Leishmania*, AIDS, HIV, mNGS, rapid diagnosis

## Abstract

*Leishmania* belongs to a genus of the protozoan parasites that causes leishmaniasis, and includes cutaneous leishmaniasis (CL) and visceral leishmaniasis (VL). In this case, *Leishmania* amastigotes were found on cytomorphology examination of the bone marrow specimen, followed by 1,076 *Leishmania donovani* reads using metagenomic next generation sequencing (mNGS). Since being definitely diagnosed with VL/HIV coinfection, the patient was treated with liposomal amphotericin B as the parasite-resistant therapy and was discharged after clinical cure. But nearly a year later, on the mNGS follow-up, *L. donovani* was detected in the patient’s blood plasma specimen with 941 reads, suggesting that a relapse of leishmaniasis had occurred. These results indicate that leishmaniasis still exists in China and may represent a public health concern. This case could be helpful in the differential diagnosis of leishmaniasis, and for determining disease progression, prevention, and control of vectors and reservoir hosts.

## Introduction

Leishmaniasis, a zoonotic disease caused by *Leishmania* that is spread by the sand fly species. It is a poverty-related disease with two main clinical forms: visceral leishmaniasis (VL) and cutaneous leishmaniasis (CL) (Palumbo, 2009). An estimated 0.9–1.3 million new cases of leishmaniasis per year are reported in nearly 100 countries and regions (Bora, 1999; Gramiccia and Gradoni, 2005; Ready, 2014). As a global public health concern, *Leishmania* mainly affects some of the poorest individuals in the world and is related to malnutrition, poor housing conditions, population displacement, lack of financial resources, and a weakened immune system (Gramiccia, 2011). VL has existed in China for at least 120 years (Han et al., 2018; Han et al., 2021). From 2002 to 2011, a total of 3,169 cases of visceral leishmaniasis were reported in China, with approximately 140 to 509 cases diagnosed per year (Lun et al., 2015). This suggests that leishmaniasis is not extinct in China, and has the potential to cause a public health problem.

As a major worldwide public health concern, the human immunodeficiency virus (HIV) infection is present in all countries and regions. There are about approximately 36.9 million individuals with HIV, and 2.0 million new infections are reported every year (Molina et al., 2003). It is clear that there is an overlap between leishmaniasis transmission and acquired immune deficiency syndrome (AIDS), resulting in an increasing number of cases of *Leishmania*–HIV coinfection (Puig and Pradinaud, 2003; Correa Soares et al., 2020).

Progress in the diagnosis of leishmaniasis depends on the development of effective methods and the discovery of suitable biomarkers. There are several approaches that have been used in the diagnosis of leishmaniasis, such as microscopic examination, culture, serologic diagnosis, and molecular approaches (Pace, 2014; von Stebut, 2017). However, these methods have similar limitations, including the non-routine availability of reagents. More importantly, physicians rarely consider the infection of such rare pathogens. Recently developed mNGS can overcome the limitations of traditional diagnostic tests. This new technology identifies all pathogens directly from the sample through a single run in a hypothesis-free and culture-independent manner. Studies have shown that mNGS provides more sensitive findings than the traditional culture method in clinical conditions such as immunodeficiency syndrome, blood stream, respiratory, and general infections (Wang et al., 2021). More importantly, due to unbiased sampling, mNGS is theoretically able to identify not only known pathogens but also unexpected pathogens and even discover novel organisms (Han et al., 2019; Zhang et al., 2020).

It should be noted that mNGS also has some limitations such as human genome contamination and possible environmental microbial contamination. The vast majority of reads obtained in mNGS are derived from the human host. This would impede the overall analytical sensitivity of mNGS for pathogen detection. Host depletion methods or targeted sequencing may help to partially mitigate this disadvantage. Because mNGS alone cannot define whether the detected microbe is the causative pathogen or environmental microorganism, a multidisciplinary approach by clinicians, microbiologists, and the lab technicians is required to interpret the result. The application of sequencing technology is shifting from research to clinical laboratories due to rapid technological developments and substantially reduced costs. Before this new approach could be considered as a first-line diagnostic test in *Leishmania* infection, the costs and availability should be further improved.

**Figure 1:**
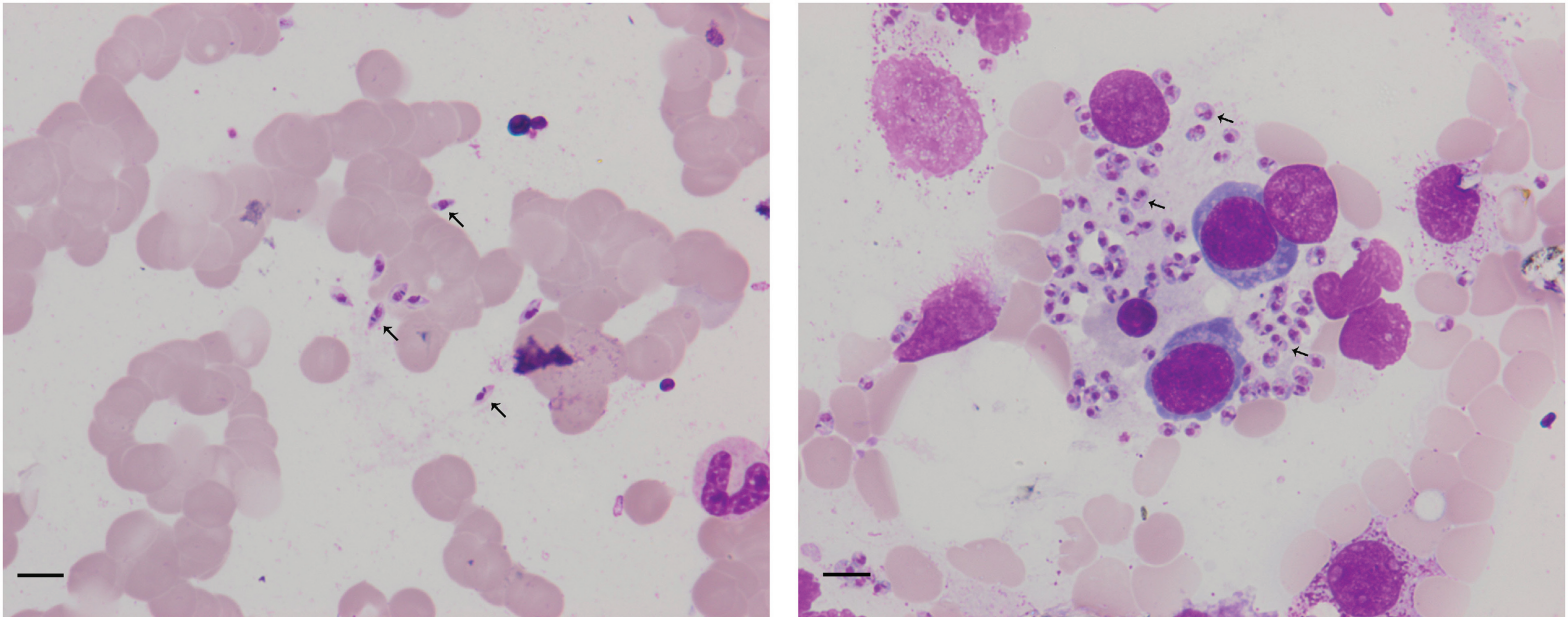
Arrowheads show *Leishmania* amastigotes in bone marrow cytology, which are oval and 2.9–5.7 × 1.8–4.0 µm in size. Scale bar = 10 μm. The cytoplasm is stained lilac or purple-blue with Wright’s stain and contains a large round nucleus. The nucleus (red or lavender) is located at the front of the worm and accounts for a third to a half of the worm’s length.

The patient in this case showed a co-infection with *Leishmania* and HIV, which was diagnosed by bone marrow microscopic examination (**Figure 1**) and mNGS. To our knowledge, this is a rare report of leishmaniasis diagnosed by mNGS in China.

## mNGS Protocol

DNA was extracted from 600 μl of bone marrow or plasma using the TIANamp Micro DNA Kit (DP316, TIANGEN BIOTECH, Beijing, China). We extracted the DNA from the negative and positive quality control sample along with the test specimens. Then, DNA libraries were constructed through DNA-fragmentation, end-repair, adapter-ligation, and PCR amplification. The constructed library was qualified by Agilent 2100 (Agilent Technologies, Santa Clara, CA) and using the Qubit 2.0 Fluorometer (Invitrogen, USA). The qualified double-strand DNA library was transformed into a single-stranded circular DNA library through DNA-denaturation and circularization. DNA nanoballs (DNBs) were generated from single-stranded circular DNA using rolling circle amplification (RCA). The DNBs were qualified using Qubit 2.0 Fluorometer. Qualified DNBs were loaded into the flow cell and sequenced (50 bp, single-end) on the BGISEQ-50 platform.

**Figure 2:**
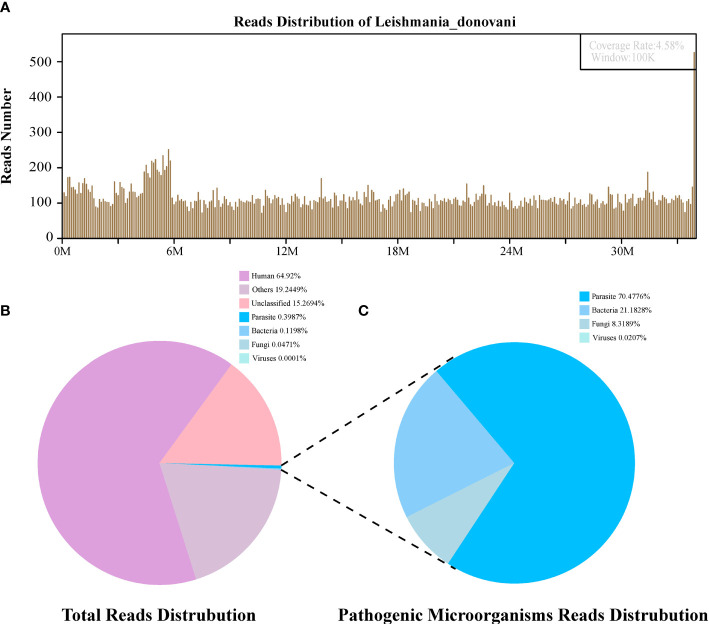
The diagnosis of *Leishmania* infection by Metagenomic next-generation sequencing (mNGS). **(A)** Mapping of *Leishmania donovani* reads on the genome. **(B)** Reads distribution of total DNA in the bone marrow sample. **(C)** Distribution of pathogenic microorganisms reads in the absence of human, others and unclassified reads.

**Figure 3:**
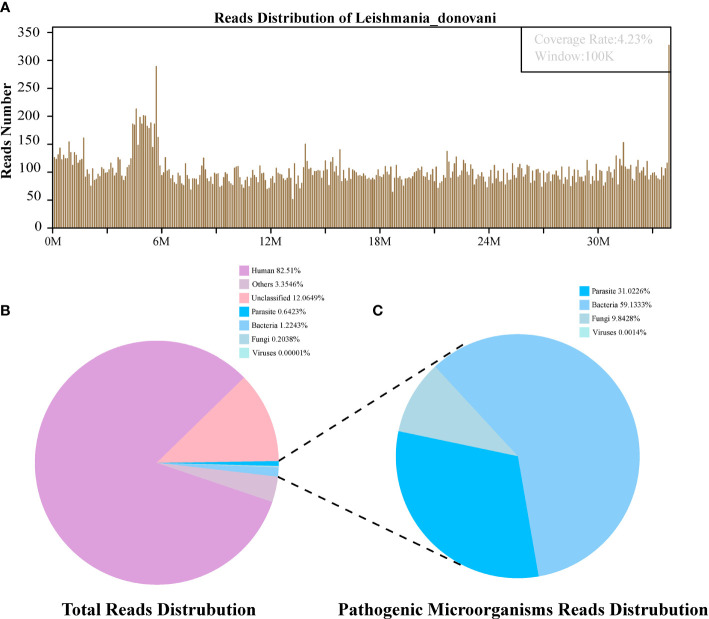
The follow-up diagnosis of *Leishmania* infection by Metagenomic next-generation sequencing (mNGS). **(A)** Mapping of *Leishmania donovani* reads on the genome. **(B)** Reads distribution of total DNA in the blood plasma sample. **(C)** Distribution of pathogenic microorganisms reads in the absence of human, others and unclassified reads.

High-quality sequencing data were generated by removing low-quality and short reads (length <35 bp) using in-house software, followed by computational subtraction of human host sequences mapped to the human reference genome (hg19) using Burrows–Wheeler Alignment. After the removal of low complexity reads, the remaining data were classified by simultaneously aligning to Pathogens metagenomics Database (PMDB), consisting of viruses, bacteria, fungi, and parasites. The four Microbial Genome Databases were downloaded from NCBI (ftp://ftp.ncbi.nlm.nih.gov/genomes/). RefSeq contains 4,945 whole-genome sequences of viral taxa, 6,350 bacterial genomes or scaffolds, 1,064 fungi, and 234 parasites associated with human diseases. The number of unique alignment reads was calculated and standardized to get the number of reads stringently mapped to pathogen species (SDSMRN) and the number of reads stringently mapped to pathogen genus (SDSMRNG). The amount of sequencing data produced by bone marrow and blood plasma mNGS after each step is shown in **Figures 2**, **3**.

## Results

An adult female was diagnosed with “HIV infection, liver cirrhosis” on admission with symptoms of fatigue and splenomegaly. A specific pathogen infection was suspected, and then the diagnosis of AID with visceral leishmaniasis was confirmed by bone marrow microscopy and mNGS. A relapse of leishmaniasis occurred in the follow-up reviews with mNGS nearly one year later.

The first sequencing detection identified 95,335 (among a total 23,910,670) sequence reads (0.3987%) matching to the parasite metagenomics database (**Figure 2B**), and the *Leishmania* genome reads (8,4831, 88.98%) were predominant among the parasites species, which corresponded to 1,076 *L. donovani* reads covering a high percentage (4.58%).Similarly, in the second detection, we obtained 145,456 (of a total 27,400,143) sequence reads belonging to the *Leishmania* genus genome, and there were 941 *L. donovani* uniquely aligned sequences corresponding to the *Leishmania* genome with a high coverage percentage (4.23%). As expected, no *Leishmania* read was detected from control sample.

## Case Descriptions

The patient, a 54-year-old woman, was admitted to the infection ward of the Liaocheng People’s Hospital, China, for “recurrent fatigue, cough, pitting edema of the lower extremities, chest tightness, and dyspnea especially after activities for two years, sustained cough for two days” in October, 2019. Upon tracing the patient’s medical history, the following pieces of information were discovered: this patient was diagnosed with “AIDS” nine years ago at the hospital of Xinjiang Province without receiving standard treatment. Four years ago, she was diagnosed with “cirrhosis” due to fatigue and received liver protection treatment at the same hospital. However, she did not take any medications after being discharged from the hospital. She has been using oral anti-HIV drugs (lamivudine, compound sulfonic acid armor oxazole piece, and tenofovir) since 2018.

Born in Liaocheng of Shandong Province, this patient resided in the forest region of Xinjiang Province for more than 10 years, and returned to Liaocheng four years ago. There was no history of smoking, drinking, or toxic exposure. The physical examination revealed the following findings: blood pressure 105/51 mmHg, splenomegaly, and slight pitting edema over both legs. The routine laboratory tests were as follows: blood samples white blood cells (WBC) 1.14 × 10^9^ cells/L, red blood cells (RBC) 1.19 × 10^9^ cells/L, hemoglobin (Hb) 27 g/L, platelets (PLT) 41 ×8·10^9^ cells/L, procalcitonin 0.34 ng/ml, C-reactive protein 15.15 mg/L; Urine samples: WBC 53 cell/ul and urobilinogen 34 μmol/L (+); Virus screening: hepatitis B surface antigen (HBs-Ag) >250 IU/ml, hepatitis B e antibody (HBe-Ab) 0.66 s/co, hepatitis B core antibody(HBC-Ab) 7.78 s/co, human immunodeficiency virus antibody(HIV-Ab) positive; Biochemical analysis: alanine aminotransferase (ALT)13 IU/L, aspartate aminotransferase (AST) 24 IU/L, albumin (ALB) 18 g/L, globulin (GLB)70 g/L, albumin/globulin (ALB/GLB) 0.26, cholinesterase (CHE) 1115 U/L, total bilirubin (TBIL) 27.2 μmol/L, direct bilirubin (DBIL) 20.1 μmol/L, uric acid (UA) 418 μmol/L, β2–macroglobulin (β2-mb) 5.29 mg/L, cystatin C (CYS-C) 1.14 mg/L, creatine kinase MB subtype(CK-MB) 24 μ/L; T cell subgroup count: CD4 23.01%, CD8 35.23%, CD4/CD8 0.65; Immunoglobulin + complement: C3 0.63 g/L, C4 0.12 g/L, IgG 44.90 g/L, IgA 5.79 g/L, IgM 15.30 g/L; Blood ammonia (AMON): 52.9 μmol/L; abdominal ultrasonography: diffuse liver disease (suggesting early liver cirrhosis), vessel broadening of the main portal vein and splenic vein, unclear wall of the gallbladder, splenomegaly; chest CT: bronchitis, pneumonia in the left lower lobe, multiple nodules in both lungs, mediastinal axillary lymph nodes; Examination of bone marrow cytomorphology: erythroid hyperplasia, higher polychromatic erythroblast, mature red blood Rouleau rank, abundant amastigotes of *L. donovani* around histiocytes (**Figure 1**); mNGS of bone marrow sample: 1,076 reads of *L. donovani* (**Table 1**).

**Table 1:** The basic situation of *Leishmania donovani* in twice NGS-based detections.

Sample	Type	Parasite identified	No. of unique reads	No. of *Leishmania* reads	No. of parasite reads	Proportion in parasite reads (%)	Coverage, (%)	Depth
First detection	Bone marrow	*Leishmania donovani*	1,076	84,831	95,335	1.1287%	4.58	1.07
Second detection	Plasma	*Leishmania donovani*	941	145,456	175,991	0.5347%	4.23	1.06

The patient was hospitalized for AIDS and liver cirrhosis, and the relevant examinations revealed that RBC, WBC, and PLT counts decreased in peripheral blood, liver damage and pneumonia. After active treatments (blood transfusion to treat anemia, albumin infusion, anti-HIV therapy, levofloxacin to treat the infection, hepatoprotection to treat jaundice, rising white cells, diuresis, and potassium supplements), these symptoms showed no signs of improvement.

According to the results of the patient’s bone marrow cytomorphology examination and sequencing analysis, the patient was diagnosed with “leishmaniasis combined with AIDS”. Therefore, in the original treatment regimen, amphotericin B was administered for parasite-resistant therapy. The treatment schedule was the first day 5 mg dose for 6 h by continuous intravenous pumping on October 18, 2019, the dose was gradually increased to 5 mg day by day. On October 23, 2019, the blood parameters showed that the white cells were lower and there were signs liver damage and renal damage. Taking into account the side effects of amphotericin B, the treatment frequency and dose were changed to 20 mg every two days in a single dose (continuous intravenous administration for 6 h) of amphotericin B. We started to use liposomal amphotericin B On 30 October, scheduled for 3 mg/kg/time from the first day to the fourth day (continuous intravenous pumping 4 h), 1.6 mg/kg on day 13 (continuous intravenous pumping 4 h), 1.6 mg/kg on day 20 (continuous intravenous pumping 4 h), totally for 6 times. Then morphological examination of the bone marrow specimen was checked, and *Leishmania* was not detected. The patient’s symptoms improved and she was discharged from the hospital.

The patient was readmitted to the hospital for treatment due to fatigue in October 2020. Considering that it might be a relapse of leishmaniasis, mNGS tests for blood plasma specimen were performed, with the consent of her family. The results showed 941 reads of *L. donovani* (**Table 1**). However, considering the high cost of antiparasitic infection, the patient received supportive treatment and was discharged after symptom reduction.

## Discussion

In this study, we describe a patient diagnosed with HIV infection, liver cirrhosis on admission to hospital with symptoms of fatigue and splenomegaly. A specific pathogen infection was suspected after collecting demographic information from the patient: a history of residing in the forest region in Xinjiang Province for more than 10 years, clinical symptoms, laboratory test results, imaging examination results, diagnosis and treatment history, and prognostic results. The diagnosis of HIV with *L. donovani* infection was definitely confirmed by mNGS (Tavares et al., 2003). In this study, we diagnosed a rare *Leishmania* infection in an HIV patient by metagenomic analysis. The patient was treated with liposomal amphotericin B and after one year of the initial treatment, mNGS detected 941 *L. donovani* reads from the patient’s plasma sample, suggesting a relapse of leishmaniasis. *Leishmania donovani* is a specific pathogen that is not normally present in the environment, serological or PCR reagents for this pathogen are not normally prepared routinely in the laboratory. This patient showed signs of infection on his second admission, so we screened him for possible infectious agents using mNGS, which eventually revealed the recurrence of leishmaniasis. Diagnosis using mNGS for *Leishmania* infection is a novel approach has raised concerns that *Leishmania* is still present in Central China. This finding provides a valuable reference for diagnosis.

Based on previous research, it can be concluded that the clinical manifestations of VL in HIV-infected patients are similar to those of non-coinfected individuals (Puig and Pradinaud, 2003). Typical VL includes intermittent or continuous fever, non-tender hepatosplenomegaly, pancytopenia, leading to anemia, hemorrhages and concurrent infections (de Araujo et al., 2012). However, the atypical form may be misdiagnosed with other opportunistic infections or is diagnosed with substantial delay, so early recognition and treatment are necessary (Nourian et al., 2019). In this case, the patient manifested unusual clinical presentations (fatigue, splenomegaly, anemia, no fever, hemorrhages, or concurrent infections), which frequently occur in individuals with low CD4+ T-cell counts (23.01%) (Bimal et al., 2008; Garg et al., 2009; Santos-Oliveira et al., 2010). Its timely etiologic diagnosis is challenging.

Some elements relevant to the host and environment can affect the prevalence of VL–HIV coinfection (Natalia Souza et al., 2018). *Leishmania* and HIV share an immunopathological mechanism, compromising macrophages and dendritic cells, which is typical of the presence of both pathogens, accelerating the progression of VL and HIV (Vassallo et al., 2014). The impaired immune function of VL/HIV coinfected patients may: (i) favor the reactivation of latent *Leishmania* infection; (ii) induce a more severe presentation of visceral Leishmaniasis; (iii) cause a poorer therapeutic response; and (iv) increase the risk of relapse after treatment (Olivier et al., 2003; Monge-Maillo and Lopez-Velez, 2016).

Therefore, the main challenges in the therapy of VL/HIV coinfection are developing an effective drug that not only resolves the first episode of VL, but also prevents relapses. However, there is rare evidence and data on the optimal therapy for VL/HIV coinfection. Liposomal amphotericin B is a new treatment with the characteristic of relatively high efficiency and low toxicity (Ponte-Sucre et al., 2017; Roatt et al., 2020). The World Health Organization (WHO, 2010) recommended liposomal amphotericin B as a first line therapy drug for visceral leishmaniasis in African countries (Abongomera et al., 2018). Ultimately, liposomal amphotericin B was used for antiparasitic therapy, and the overall treatment outcome was successful. Side effects emerged involving liver and kidney injury, which were significantly improved by a timely adjustment of the treatment schedule.

As shown previously, leishmaniasis–HIV coinfection presents some difficulties for diagnosis and identification due to atypical clinical manifestations, both in visceral and cutaneous forms. Additional toxicity should be avoided in the treatment of *leishmania*–HIV coinfection, and minimal side effects should be sought. Liposomal amphotericin B is the main drug for the treatment of leishmaniasis. However, dosing is still a significant problem, as there is no consensus on the optimal treatment regimen for different individuals worldwide (Silva Ferreira de Carvalho et al., 2020). An important point to be explored is the search for new active drugs against all species of *Leishmania*. After the clinical cure is obtained, parasites continue to be present in some organs or lesions, and reactivations or relapses of leishmaniasis may occur if immunosuppression is still present.

## Data Availability Statement

The datasets for this article are not publicly available due to concerns regarding participant/patient anonymity. Requests to access the datasets should be directed to the corresponding author.

## Ethics Statement

Written informed consent was obtained from the individual(s) for the publication of any potentially identifiable images or data included in this article.

## Author Contributions

PS, SC, and CW performed bioinformatics analysis. PS participated in the writing of the manuscript. XT collected cases. YG, JF, and ZY performed mNGS testing. CW analysed and visualised the data. QZ and FP conceived the study and participated in the writing of the manuscript. All authors contributed to the article and approved the submitted version.

## Funding

This study was supported by the Medical Science and Technology Development Foundation of Shandong Province, China (Grant No. 2019WS113).

## Conflict of Interest

The authors declare that the research was conducted in the absence of any commercial or financial relationships that could be construed as a potential conflict of interest.

## Publisher’s Note

All claims expressed in this article are solely those of the authors and do not necessarily represent those of their affiliated organizations, or those of the publisher, the editors and the reviewers. Any product that may be evaluated in this article, or claim that may be made by its manufacturer, is not guaranteed or endorsed by the publisher.
